# Driving Status in Patients Admitted to Acute Psychiatric Ward and DVLA Advice

**DOI:** 10.1192/bjo.2024.599

**Published:** 2024-08-01

**Authors:** Kerenza MacDaid, Faryal Rana, Reuben Tagoe

**Affiliations:** Surrey and Borders Partnership, Guildford, United Kingdom

## Abstract

**Aims:**

DVLA guidance is very clear about patients not driving during or shortly after episodes of acute mental illness. There is an obligation for patients to inform the DVLA if they are unwell. The obligation for doctors to inform the DVLA if the patient chooses not to, and continues to drive when they should not, is also well known.

This audit aims:

1. To identify the number of patients whose driving status was recorded following their admission to an acute psychiatric ward.
2. To identify the number of patients discharged with correct DVLA compliant advice.
3. To identify the number of patients whose notes reflected correct driving status information on discharge.

**Methods:**

Patient ward notes and discharge summary documents relating to their admission on to the PICU ward were examined retrospectively for recorded evidence of patient's driving status and any documented DVLA advice given. Patients admitted from November 2022 and April 2023 were reviewed. 68 patients were identified and systematic sampling techniques identified a sample of 30 patients.

Keyword search included “Driving”, “License”, “Car”, “Driving license”, “DVLA”.

**Results:**

30 patients were reviewed in total.

40% of sample patients had no driving status recorded on their notes.

Of the 60% of sample patients who were confirmed to be driving/held license, nearly half (47%) had no recorded advice documented regarding the DVLA or driving after an acute MH illness on discharge.

A third (33%) of sample patients were recorded as having been given generic advice regarding driving only.

Only 20% of sample patients recorded to be driving, were documented as having been given correct advice as per DVLA guidance on discharge.

**Conclusion:**

This audit demonstrated that driving status is currently poorly recorded in patients admitted to PICU and documentation of correct DVLA-compliant driving advice being given on discharge to relevant patients is also poor. Patients may not be receiving important information that they need.

Providing correct and accurate advice to patients regarding the DVLA rules and psychiatric illness should be part of a safe and robust discharge plan, and forms part of the clinical teams obligations to the patient. Identifying patients as drivers and improved documentation of driving status and evidencing appropriate advice being given is key.

A number of interventions were implemented and a re-audit will be undertaken in Spring 2024. If successful at improving rates of DVLA compliant advice being given, it would be hoped these interventions could be shared across the trust.

